# Soft Palate Perforation with Endotracheal Tube During Video Laryngoscopy

**DOI:** 10.5811/cpcem.64731

**Published:** 2026-08-31

**Authors:** Samuel J. Thomas, David I. Zimmer, Jack H. Langford, Toby J. Brenner, Kate M. Kelly, Donald F. Zimmer

**Affiliations:** * Indiana University School of Medicine, Department of Emergency Medicine, South Bend, Indiana; † University of Louisville Hospital, Department of Otolaryngology, Louisville, Kentucky; ‡ George Washington University School of Medicine and Health Sciences, Department of Clinical Research and Leadership, Washington, District of Columbia; § Memorial Hospital, Department of Emergency Medicine, South Bend, Indiana

**Keywords:** endotracheal intubation, laryngoscopy, soft palate, oropharynx, soft tissue injuries

## Abstract

**Case presentation:**

A 57-year-old man with a history of obesity-hypoventilation syndrome, diabetes mellitus, and chronic renal insufficiency was intubated with an 8.0-mm endotracheal tube under video laryngoscopy in the emergency department due to respiratory failure. After successful intubation, bloody secretions were noted in the patient’s mouth. Subsequent examination revealed that the endotracheal tube had caused a through-and-through perforation at the junction of the soft palate and the anterior tonsillar pillar. Because the intubation had been successful, the endotracheal tube was not removed, and the soft palate was transected to free the tube. The patient made an uneventful recovery.

**Discussion:**

Soft palate perforation is an uncommon injury that has been reported more frequently with video laryngoscopy than direct laryngoscopy. To prevent this injury and ensure its rapid recognition, it is recommended that the clinician sequentially look at the “5 M’s” during intubation: Mouth–Monitor–Mouth–Monitor–Mouth.

## CASE PRESENTATION

A 57-year-old man with a past medical history significant for obesity-hypoventilation syndrome, diabetes mellitus, and chronic renal insufficiency presented to the emergency department (ED) in respiratory failure. The vital signs were as follows: respiratory rate, 32 breaths per minute; blood pressure, 90/60 mm Hg; pulse, 130 beats per minute; temperature, 98.6 °F; and oxygen saturation was 83% with diminished bilateral breath sounds, which led to the patient being placed on bilevel positive airway pressure. After 30 minutes, arterial blood gases revealed the following: pH 7.06 (reference range, 7.35–7.45); oxygen pressure, 47 mm Hg (75–105 mm Hg); and carbon dioxide pressure, 116 mm Hg (33–45 mm Hg). The patient therefore required endotracheal intubation, which was performed using a video laryngoscope with a Macintosh 4 blade. The first attempt failed because of gastric secretions that required suctioning. The second attempt was successful; however, the secretions were noted to be bloody (Image).

Immediate end-tidal carbon dioxide detection was normal when the patient was placed on synchronized intermittent mandatory ventilation. He was transferred to the intensive care unit. Continued bleeding from the oropharynx resulted in an otolaryngology consultation. Evaluation of the oropharynx revealed a right soft palate perforation through which the endotracheal tube (8.0-mm inner diameter, 11.8-mm outer diameter) passed directly into the trachea (Video). Because the patient was ventilated satisfactorily, the endotracheal tube was repositioned anteriorly and medially, allowing for transection and repair of the soft palate. He made an uneventful recovery following five days of mechanical ventilation, during which he responded to diuresis, pulmonary toilet, bronchodilators, and corticosteroids.

## DISCUSSION

Recent literature demonstrates that videolaryngoscopy–guided endotracheal intubation improves first-pass success rates and laryngeal views. Consequently, this approach has become the default technique in EDs and operating rooms.^1^,^2^ However, injuries to the soft palate and surrounding structures occur more frequently with video laryngoscopy than with direct laryngoscopy.^3^,^4^ This case report describes an uncommon injury to the soft palate and anterior tonsillar pillar during endotracheal intubation.

A mnemonic designed to improve accuracy and prevent complications during video laryngoscopy–guided intubation is the “Mouth–Screen–Mouth–Screen” sequence.^5^ However, this technique does not emphasize reevaluation of the mouth after intubation. Therefore, a more detailed mnemonic is recommended to prompt clinicians to look at the “5 M’s” (Mouth–Monitor–Mouth–Monitor–Mouth: (1) Look into the patient’s mouth while inserting the laryngoscope blade; (2) look at the monitor display of the glottis to position the camera; (3) look again into the mouth to insert the endotracheal tube and avoid perforations with the rigid stylet; (4) look at the monitor to position the tube before popping the stylet, advancing through the vocal cords, and withdrawing the stylet once the tube is in its proper position; and (5) look directly at the mouth while removing the laryngoscope to evaluate for any injuries while ensuring the tube is secured.

*CPC-EM Capsule*

What do we already know about this clinical entity?

*Perforations and other injuries to the soft palate during endotracheal intubation are uncommon complications that are reported more often during video laryngoscopy.*

What is the major impact of the image(s)?

*The image and video show the location of the perforation with the endotracheal tube caught in the soft palate and relevant labels to help orient the viewer.*

How might this improve emergency medicine practice?

*The proposed mnemonic Mouth–Monitor–Mouth–Monitor–Mouth indicates where clinicians should look to prevent and identify injuries during video laryngoscopy.*

## Supplementary Information

Video This video shows the perforation at the junction of the soft palate and the right anterior tonsillar pillar by the endotracheal tube.

## Figures and Tables

**Image:**
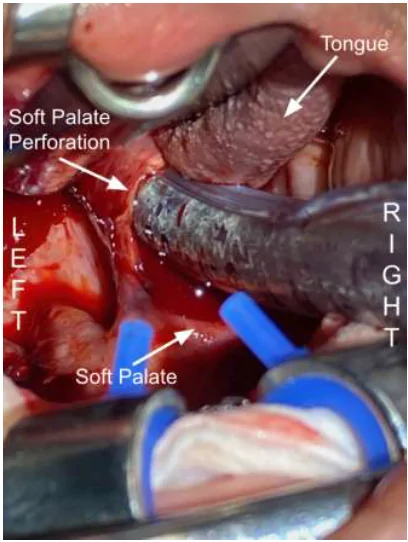
Endotracheal tube perforating the soft palate.
